# Convenient method for preparing benzyl ethers and esters using 2-benzyloxypyridine

**DOI:** 10.3762/bjoc.4.44

**Published:** 2008-11-26

**Authors:** Susana S Lopez, Gregory B Dudley

**Affiliations:** 1 Department of Chemistry and Biochemistry, Florida State University Tallahassee, FL 32306-4390 USA. Fax: (850) 644-8281

**Keywords:** alcohols, alkylation, benzyl, electrophilic substitution, esters, ethers, protecting groups, reagent

## Abstract

2-Benzyloxy-1-methylpyridinium triflate (**1**) is emerging as a mild, convenient, and in some cases uniquely effective new reagent for the synthesis of benzyl ethers and esters. This article provides a revised benzyl transfer protocol in which N-methylation of 2-benzyloxypyridine delivers the active reagent in situ. Observations on the appropriate choice of solvent (toluene vs. trifluorotoluene) and the extension of this methodology to the synthesis of other arylmethyl ethers are included.

## Introduction

As organic and medicinal chemists tackle synthetic targets of ever increasing complexity [1], the need for specialized reagents [2] and protecting groups [3–4] increases. Few protecting groups are as widely used as the benzyl (Bn) group, but protection of complex alcohol substrates as benzyl ethers is often frustrated by the need to employ basic or acidic conditions that may not be compatible with intricate systems.

**Figure 1:**
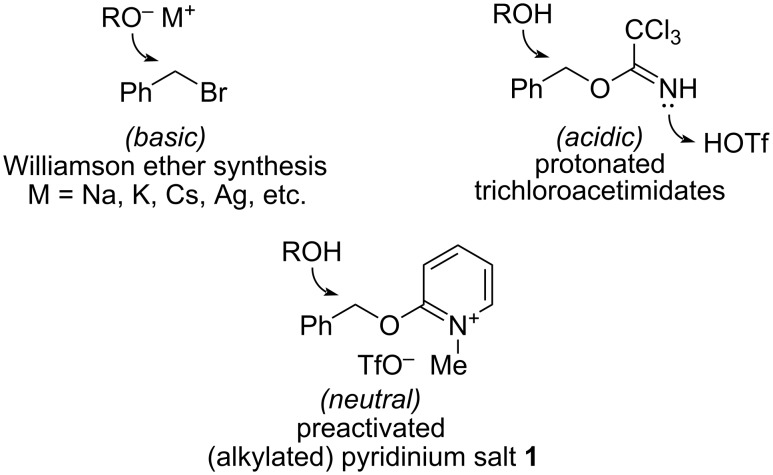
Benzyl bromide, benzyl trichloroacetimidate, and 2-benzyloxy-1-methylpyridinium triflate (**1**).

**Scheme 1:**
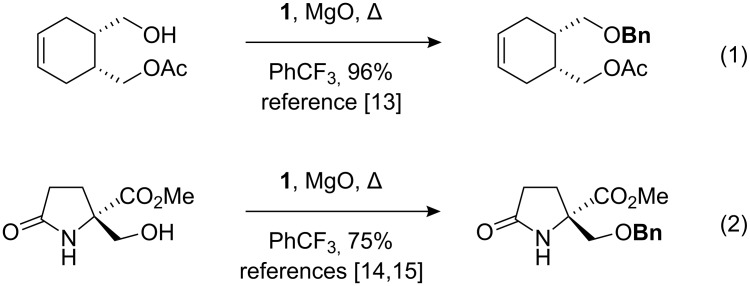
Published syntheses of benzyl esters from alcohols using neutral reagent **1**; other benzylation procedures were not successful.

Reagents that can install protecting groups under neutral conditions find immediate use in chemical synthesis [5]. 2-Benzyloxy-1-methylpyridinium triflate (**1**, Figure 1) is one such reagent [6–7]. This neutral organic salt mirrors the reactivity of benzyl trichloroacetimidate [8–11], but it does not require acidic conditions for activation [12]. Benzyloxypyridinium **1** releases an electrophilic benzyl species upon warming; application to the synthesis of benzyl ethers from alcohols for which other protocols were unsuitable has been demonstrated independently (eq 1 [13] and 2 [14–15] in Scheme 1).

N-Methylation of 2-benzyloxypyridine (**2**) furnishes crystalline **1**, which is collected by filtration and may be stored for later use [16–18]. For routine and repeated use, isolation and storage of **1** is most convenient. Alternatively, in situ activation of **2** without isolation of the active salt presents certain advantages, such as described for the synthesis of PMB ethers [19].

Herein we report new reaction protocols that build on recent reports from this laboratory [6–7,16,20] and provide the following new observations:

1. Benzyl ethers can be prepared in good to excellent yield by in situ methylation of 2-benzyloxypyridine in the presence of alcohols and magnesium oxide.
2. This simple protocol extends to the synthesis of other arylmethyl ethers and esters.
3. Toluene is a suitable solvent for most applications, although trifluorotoluene is required in at least one case.
4. 2-Benzyloxypyridine is conveniently prepared, now without using 18-crown-6.

2-Benzyloxypyridine serves as a surrogate of (or replacement for) benzyl trichloroacetimidate: alkylation of 2-benzyloxypyridine with methyl triflate provides an active reagent similar to the species produced by protonation of benzyl trichloroacetimidate using triflic acid, except that alkylation under neutral conditions is compatible with acid- (and base-) sensitive substrates.

## Results and Discussion

**Scheme 2:**
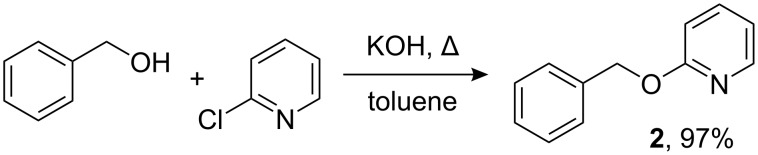
Preparation of 2-benzyloxypyridine (**2**).

2-Benzyloxypyridine was prepared in 97% yield by heating a mixture of benzyl alcohol, 2-chloropyridine (1.1 equiv), and solid potassium hydroxide at reflux in toluene for 1 h (Scheme 2). This protocol differs slightly from those previously reported [16,21], which included 18-crown-6 (5 mol%); omission of 18-crown-6 simplifies the process.

For the synthesis of benzyl ethers, a mixture of the alcohol substrate (**3**), 2-benzyloxypyridine (**2**), and magnesium oxide in toluene was cooled to 0 °C and treated with methyl triflate. The reaction mixture was allowed to warm to room temperature and then heated at 90 °C for 24 h. Table 1 summarizes the results from the benzylation of a representative group of functionalized alcohols under these new conditions (Method A), as well as results obtained under the previously reported conditions using pre-formed pyridinium salt **1** and trifluorotoluene as the solvent (Method B, entries 2, 4, and 6).

**Table 1:** Benzylation of representative alcohols promoted by N-methylation of 2-benzyloxypyridine (**2**).

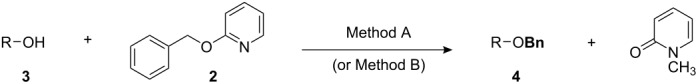				
Entry	R–OH	Method	R–OBn	Yield

1 2	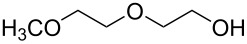 **3a**	A^a^ B^b^	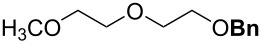 **4a**	92% 93% [6]
3 4	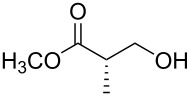 **3b**	A B	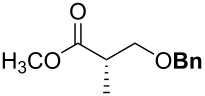 **4b**	79% 76–82% [16]
5 6	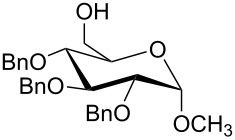 **3c**	A B	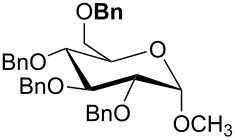 **4c**	0%^c,d^; 93%^d,e^ 84%^d^
7	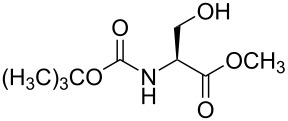 **3d**	A	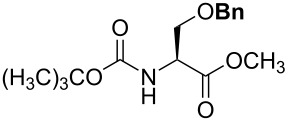 **4d**	84% [24]
8	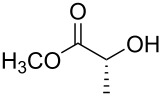 **3e**	A	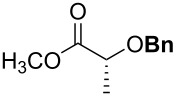 **4e**	79%^d,e^

^a^Method A (in situ formation of **1**): 1.0 equiv alcohol, 2.0 equiv **2**, 2.0 equiv MgO, toluene, mix at 0 °C, then add 2.0 equiv MeOTf, then heat mixture at 90 °C for 24 h. ^b^Method B (pre-formed **1**): 1.0 equiv alcohol, 2.0 equiv **1**, 2.0 equiv MgO, trifluorotoluene, 85 °C, 24 h. ^c^No reaction in toluene despite repeated attempts (starting material recovered unchanged). ^d^3.0 equiv of each reagent. ^e^Trifluorotoluene used as the solvent.

Benzylations of monoglyme (**3a**) and Roche ester (**3b**) were accomplished with similar efficiency whether the active reagent **1** was formed in situ (entries 1 and 3) or isolated prior to use (entries 2 and 4). Glucose derivative **3c** failed to react in toluene, but switching the solvent to trifluorotoluene restored reactivity (entry 5, 93%). Toluene is a cheaper and more common solvent than trifluorotoluene, but toluene has a lower dipole moment and also is subject to Friedel–Crafts benzylation under the reaction conditions [6,22]. Trifluorotoluene (also known as benzotrifluoride or BTF) is recommended as a “green” solvent alternative to dichloromethane [23]. Benzylation reactions of *N*-Boc-serine **3d** (entry 7, 84%) and methyl lactate (**3e**, 79%) verify compatibility with esters and carbamates. Note that the benzylation of *N*-Boc-serine methyl ester (**3d**) compares favourably to analogous reactions reported previously [24], because the neutral reaction conditions described herein are compatible with the acid-labile Boc group and the base-labile β-hydroxy ester.

**Scheme 3:**
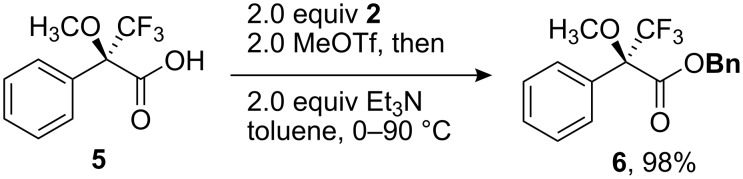
Synthesis of a benzyl ester from a carboxylic acid.

Minor modification of the above procedure renders it suitable for the formation of benzyl esters from carboxylic acids (Scheme 3). In order to avoid the potential for competing N-methylation of triethylamine, which is the optimal acid scavenger for the benzylation of carboxylic acids [20], methyl triflate was added to a toluene solution of Mosher’s acid **5** and 2-benzyloxypyridine (**2**) prior to addition of triethylamine. Heating the resulting mixture for 24 h furnished benzyl ester **6** in 98% yield.

**Scheme 4:**
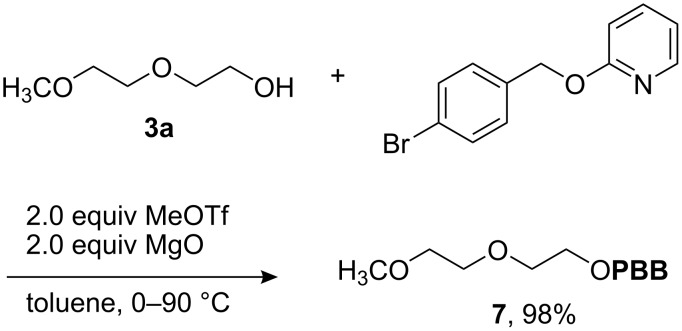
Representative synthesis of a halobenzyl ether under neutral conditions.

Neutral, isolable pyridinium triflate salts are suitable for the synthesis of halobenzyl ethers [25], which are emerging from their niche in natural products synthesis [26] because of their growing importance in carbohydrate chemistry [27–31]. The experiment outlined in Scheme 4 suggests that the observations described in this article for the synthesis of benzyl ethers are equally relevant for the synthesis of halobenzyl ethers (**3a** → **7**, 98% yield).

Finally, replacement of methyl triflate with less reactive methylating reagents, including dimethyl sulfate (Me_2_SO_4_), methyl tosylate (MeOTs), and methyl iodide (MeI), did not provide comparable results. N-Methylation of 2-benzyloxypyridine with methyl triflate occurs at or below room temperature to furnish triflate salt **1** in a manner of minutes. In contrast, reaction of methyl iodide with 2-chloropyridine requires heating in acetone at reflux for 24 h [32] to provide Mukaiyama’s reagent [33–34] (2-chloro-1-methylpyridinium iodide). If release of the reactive benzyl electrophile (which begins to occur upon warming) competes with N-methylation of pyridine, then side reactions and alternative pathways emerge. For example, Paquette and co-workers report that the use of methyl tosylate in a related system is associated with sulfonic acid-catalyzed arylmethylation reactions [35]. Therefore, methyl triflate is recommended for promoting the arylmethylation of alcohols under the neutral, magnesium oxide-buffered conditions reported herein.

## Conclusion

A new protocol for the synthesis of benzyl ethers is described using 2-benzyloxypyridine and methyl triflate in lieu of benzyl trichloroacetimidate and triflic acid. N-Methylation of 2-benzyloxypyridine gives rise to an active benzyl transfer reagent (**1**) in situ, presumably in much the same way as N-protonation activates benzyl trichloroacetimidate. Methyl triflate can be added directly to the reaction mixture because N-methylation of pyridine is faster than methylation of the neutral alcohol. Toluene is an appropriate solvent for most applications, although trifluorotoluene is generally preferred, and trifluorotoluene was uniquely effective in one case. Proof-of-concept experiments indicate that this methodology applies equally to the synthesis of other arylmethyl ethers and esters. This new protocol is ideal when one does not wish to isolate and store reagent **1**, such as for infrequent use or rapid screening of alternative benzylation protocols.

## Experimental

**2-Benzyloxypyridine (2)**: A mixture of benzyl alcohol (11.7 g, 0.108 mol, 1.0 equiv), 2-chloropyridine (13.5 g, 0.119 mol, 1.1 equiv), KOH (20.0 g, 0.356 mol, 3.3 equiv, ground with a mortar and pestle), and anhydrous toluene (210 mL) was heated at reflux (bath temperature: 130 °C) for 1 h with azeotropic removal of water to provide 19.3 g (97%) of 2-benzyloxypyridine after aqueous workup and distillation (bp 93–95 °C, 1.0 mmHg). Inclusion of 18-crown-6 (5 mol%) in the reaction mixture afforded similar results, as described previously in a more detailed procedure [16].

**General Procedure for preparation of benzyl (arylmethyl) ethers (3 → 4)**: A mixture of alcohol **3** (1.0 equiv), 2-benzyloxypyridine (**2**, 2.0 equiv), and MgO (2.0 equiv) in toluene (10 mL per mmol **3**) was cooled in an ice bath, and methyl triflate (2.0 equiv) was added dropwise. The ice bath was replaced with an oil bath, which was gradually warmed to 90 °C and maintained at that temperature for 24 h. The reaction mixture was then allowed to cool to ambient temperature, filtered through Celite^®^ with the aid of CH_2_Cl_2_, and concentrated under reduced pressure. Purification on silica gel provided ether **4** as described in Table 1. All compounds provided spectroscopic data in agreement with literature reports.

**Mosher’s acid, benzyl ester 6**: A solution of Mosher’s acid **5** (0.072 g, 0.31 mmol, 1.0 equiv) and 2-benzyloxypyridine (0.12 g, 0.62 mmol, 2.0 equiv) in toluene (5 mL) was cooled at 0 °C. Methyl triflate (0.070 mL, 0.62 mmol, 2.0 equiv) was added dropwise, followed by triethylamine (0.085 mL, 0.62 mmol, 2.0 equiv). The resulting mixture was allowed to warm to ambient temperature and then heated at 90 °C for 24 h. After cooling to ambient temperature, the reaction mixture was filtered through Celite^®^ with the aid of CH_2_Cl_2_ and concentrated under reduced pressure. Purification on silica gel provided 0.098 g of ester **6** (98%) [20].
